# Dissecting the role of guanidine copper complexes in atom transfer radical polymerization by density functional theory

**DOI:** 10.1186/1758-2946-3-S1-P28

**Published:** 2011-04-19

**Authors:** S Herres-Pawlis, R Haase, O Bienemann

**Affiliations:** 1 Fakultät Chemie, Technische University of Dortmund, 44221 Dortmund, Germany; 2 Department of Chemistry, University of Paderborn, 33098 Paderborn, Germany

## 

Since the development of the living / controlled radical polymerization method ATRP (= atom transfer radical polymerization) in 1995 [1] new catalysts for this reaction have been intensively investigated. This method conquered rapidly numerous fields in chemistry ranging from organic and polymer synthesis to materials science and nanotechnology.

**Figure 1:**
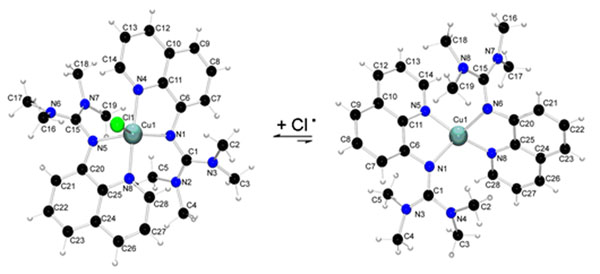
Activator/deactivator equilibrium in ATRP.

Guanidine copper complexes display high activity in ATRP of styrene but the factors imposed on the activator/deactivator equilibrium are multifaceted [2]. Herein we report on new copper complexes with the guanidine ligand 1,3,3-tetramethyl-2-(quinolin-8-yl)guanidine which produce polystyrene with a narrow weight distribution and in high yields. Kinetic studies showed that the polymerization is of living character. Structural characterization leads us to a proposal for the activator and deactivator structures which control the ATRP (Figure 1). By density functional theory, we were able to dissect the influences on the position of the equilibrium between the Cu(I) and Cu(II) complex (ligand donor strength, halogene bond strength, redox potential, coordinative space) operating the polymerization process.
